# The outcomes measured and reported in observational studies of incidental and untreated intracranial meningioma: A systematic review

**DOI:** 10.1093/noajnl/vdae042

**Published:** 2024-03-19

**Authors:** Christopher P Millward, Abdurrahman I Islim, Terri S Armstrong, Heather Barrington, Sabrina Bell, Andrew R Brodbelt, Helen Bulbeck, Linda Dirven, Paul L Grundy, Mohsen Javadpour, Sumirat M Keshwara, Shelli D Koszdin, Anthony G Marson, Michael W McDermott, Torstein R Meling, Kathy Oliver, Puneet Plaha, Matthias Preusser, Thomas Santarius, Nisaharan Srikandarajah, Martin J B Taphoorn, Carole Turner, Colin Watts, Michael Weller, Paula R Williamson, Gelareh Zadeh, Amir H Zamanipoor Najafabadi, Michael D Jenkinson, EORTC BTG, ICOM, EANO, SNO, RANO-PRO, BNOS, SBNS, BIMS, TBTC, International Brain Tumour Alliance, Brainstrust, and Brain Tumour Foundation of Canada, Kenneth Aldape, Abdurrahman I Islim, Karolyn Au, Jill Barnhartz-Sloan, Wenya Linda Bi, Felix Behling, Priscilla K Brastianos, Chaya Brodie, Nicholas Butowski, Carlos Carlotti, Ana Castro, Aaron Cohen-Gadol, Marta Couce, Michael D Cusimano, Francesco DiMeco, Katharine Drummond, Ian F Dunn, Craig Erker, Michelle Felicella, Daniel M Fountain, Evanthia Galanis, Norbert Galldiks, Caterina Giannini, Roland Goldbrunner, Brent Griffith, Rintaro Hashizume, C Oliver Hanemann, Christel Herold-Mende, Luke Hnenny, Craig Horbinski, Raymond Y Huang, David James, Michael D Jenkinson, Christine Jungk, Gerhard Jungwirth, Timothy J Kaufmann, Boris Krischek, Sylvia Kurz, Daniel Lachance, Christian Lafougère, Katrin Lamszus, Ian Lee, Jeff C Liu, Serge Makarenko, Tathiana Malta, Yasin Mamatjan, Alireza Mansouri, Christian Mawrin, Michael McDermott, Christopher P Millward, Jennifer Moliterno-Gunel, Andrew Morokoff, David Munoz, Farshad Nassiri, Houtan Noushmehr, Ho-Keung Ng, Arie Perry, Farhad Pirouzmand, Laila M Poisson, Bianca Pollo, Aditya Ragunathan, David Raleigh, Mirjam Renovanz, Franz Ricklefs, Felix Sahm, Andrea Saladino, Antonio Santacroce, Thomas Santarius, Jens Schittenhelm, Christian Schichor, David Schultz, Nils O Schmidt, Warren Selman, Helen Shih, Andrew Sloan, Julian Spears, Matija Snuderl, James Snyder, Suganth Suppiah, Erik Sulman, Ghazaleh Tabatabai, Marcos Tatagiba, Marco Timmer, Daniela Tirapelli, Joerg C Tonn, Derek Tsang, Michael A Vogelbaum, Andreas von Deimling, Tobias Walbert, Simon Walling, Justin Wang, Patrick Y Wen, Manfred Westphal, Adriana M Workewych, Stephen Yip, Gabriel Zada, Gelareh Zadeh, Viktor Zherebitskiy

**Affiliations:** Institute of Systems, Molecular, & Integrative Biology, University of Liverpool, Liverpool, UK; Department of Neurosurgery, The Walton Centre NHS Foundation Trust, Liverpool, UK; Institute of Systems, Molecular, & Integrative Biology, University of Liverpool, Liverpool, UK; Department of Neurosurgery, The Walton Centre NHS Foundation Trust, Liverpool, UK; Neuro-Oncology Branch, Center for Cancer Research, National Cancer Institute, Bethesda, Maryland, USA; Institute of Population Health, University of Liverpool, Liverpool, UK; The Brain Tumour Charity, Hampshire, UK; Institute of Systems, Molecular, & Integrative Biology, University of Liverpool, Liverpool, UK; Department of Neurosurgery, The Walton Centre NHS Foundation Trust, Liverpool, UK; Brainstrust – The Brain Cancer People, Isle of Wight, UK; Department of Neurology, Leiden University Medical Center, Leiden, The Netherlands; Department of Neurology, Haaglanden Medical Center, The Hague, The Netherlands; Department of Neurosurgery, University Hospital Southampton, Southampton, UK; National Centre for Neurosurgery, Beaumont Hospital, Dublin, Ireland; Institute of Systems, Molecular, & Integrative Biology, University of Liverpool, Liverpool, UK; Department of Neurosurgery, The Walton Centre NHS Foundation Trust, Liverpool, UK; Veterans Affairs Healthcare System, Palo Alto, California, USA; Institute of Systems, Molecular, & Integrative Biology, University of Liverpool, Liverpool, UK; Department of Neurology, The Walton Centre NHS Foundation Trust, Liverpool, UK; Division of Neuroscience, Florida International University, Miami, Florida, USA; Department of Neurosurgery, Copenhagen University Hospital, Copenhagen, Denmark; International Brain Tumour Alliance, Tadworth, UK; Nuffield Department of Surgical Sciences, University of Oxford, Oxford, UK; Division of Oncology, Department of Medicine, Medical University of Vienna, Vienna, Austria; Department of Neurosurgery, Addenbrooke’s Hospital & University of Cambridge, Cambridge, UK; Institute of Systems, Molecular, & Integrative Biology, University of Liverpool, Liverpool, UK; Department of Neurosurgery, The Walton Centre NHS Foundation Trust, Liverpool, UK; Department of Neurology, Leiden University Medical Center, Leiden, The Netherlands; Department of Neurology, Haaglanden Medical Center, The Hague, The Netherlands; Department of Neurosurgery, Addenbrooke’s Hospital & University of Cambridge, Cambridge, UK; Institute of Cancer and Genomic Sciences, University of Birmingham, Birmingham, UK; Department of Neurology, University Hospital and University of Zurich, Zurich, Switzerland; Institute of Population Health, University of Liverpool, Liverpool, UK; Department of Surgery, University of Toronto, Toronto, Ontario, Canada; Department of Ophthalmology, Leiden University Medical Centre, Haaglanden Medical Center, Haga Teaching Hospitals, Leiden and The Hague, The Netherlands; Institute of Systems, Molecular, & Integrative Biology, University of Liverpool, Liverpool, UK; Department of Neurosurgery, The Walton Centre NHS Foundation Trust, Liverpool, UK

**Keywords:** COMET, Core Outcome Set, incidental, meningioma, outcomes

## Abstract

**Background:**

The clinical management of patients with incidental intracranial meningioma varies markedly and is often based on clinician choice and observational data. Heterogeneous outcome measurement has likely hampered knowledge progress by preventing comparative analysis of similar cohorts of patients. This systematic review aimed to summarize the outcomes measured and reported in observational studies.

**Methods:**

A systematic literature search was performed to identify published full texts describing active monitoring of adult cohorts with incidental and untreated intracranial meningioma (PubMed, EMBASE, MEDLINE, and CINAHL via EBSCO, completed January 24, 2022). Reported outcomes were extracted verbatim, along with an associated definition and method of measurement if provided. Verbatim outcomes were de-duplicated and the resulting unique outcomes were grouped under standardized outcome terms. These were classified using the taxonomy proposed by the “Core Outcome Measures in Effectiveness Trials” (COMET) initiative.

**Results:**

Thirty-three published articles and 1 ongoing study were included describing 32 unique studies: study designs were retrospective *n* = 27 and prospective *n* = 5. In total, 268 verbatim outcomes were reported, of which 77 were defined. Following de-duplication, 178 unique verbatim outcomes remained and were grouped into 53 standardized outcome terms. These were classified using the COMET taxonomy into 9 outcome domains and 3 core areas.

**Conclusions:**

Outcome measurement across observational studies of incidental and untreated intracranial meningioma is heterogeneous. The standardized outcome terms identified will be prioritized through an eDelphi survey and consensus meeting of key stakeholders (including patients), in order to develop a Core Outcome Set for use in future observational studies.

**Key Points**

• Outcomes measured in meningioma observational studies are highly heterogeneous.
• Fifty-three standardized outcome terms were created from 268 verbatim outcome terms extracted.
• These will be prioritized through an eDelphi survey and consensus meeting to define a Core Outcome Set.

**Importance of the Study**

There is increasing interest in the clinical management of patients with incidental and untreated intracranial meningioma. Guidelines recommend interval MRI monitoring as first-line management but details surrounding interval timing, follow-up duration, and treatment indications are lacking. Observational research has provided limited evidence for current strategies. Variation in outcome measurement makes data comparisons difficult. In this methodological review, we have systematically identified relevant observational research, extracted outcomes measured, and applied standardized outcome terms to those with similar meaning and context. The standardized outcome terms will be prioritized through an eDelphi survey and consensus meeting of key stakeholders (including patients) in a subsequent step. This novel approach paves the way for the development of a Core Outcome Set (COSMIC: Observation) for use in future observational studies for this patient cohort. This work is one-half of The COSMIC Project (Development of Core Outcome Sets for Meningioma in Clinical Studies).

Meningiomas account for approximately one-third of all primary tumors of the central nervous system (CNS) and have increasing incidence with age (57.3 per 100 000 in adults over the age of 85).^1^ Median age at diagnosis is 66 years and females are affected more often than males (12.4 vs. 5.5 per 100 000 population).^1^ Meningiomas are classified according to The World Health Organization (WHO) Classification of Tumours of the Central Nervous System into 3 grades and 15 histopathological subtypes, with the most recent version incorporating molecular markers for the first time.^2^ Most (80.4%) are benign (WHO grade 1), while 17.9% are atypical (WHO grade 2) and 1.6% malignant (WHO grade 3).^2^

Ionizing radiation and exposure to high-dose cyproterone acetate are well-established environmental risk factors for the development of meningioma, while the most common genetic predisposing condition is NF2-schwannomatosis.^3–5^ In the absence of tumor-related symptoms and the aforementioned risk factors, a meningioma is considered to be an incidental finding. In a meta-analysis of incidental brain imaging findings from 16 studies and nearly 20 000 patients, incidental meningioma accounted for 15% of all incidental findings and the overall number needed to scan to identify an incidental meningioma was 345.^6^ The prevalence of incidental meningioma is estimated at 5 per 1000 persons.^7^ Incidentally discovered asymptomatic meningioma accounts for 20% of all newly diagnosed meningioma.^8^ Symptom development during follow-up for patients with an incidental meningioma is estimated to be 0%–8%; however, the risk of growth is estimated to be between 10% and 70%.^9,10^

International consensus guidelines currently recommend interval MRI monitoring as the first-line management strategy for an incidental meningioma; however, details surrounding interval timing, follow-up duration, and treatment indications are lacking.^11^ Subsequently, there exists heterogeneity in imaging use which leads to management decisions recommended to patients varying between active long-term MRI and clinical monitoring or upfront treatment with surgery or radiotherapy.^12^ The balance of the risks and benefits of active surveillance versus upfront treatment is not well defined.^13^

Clinical studies of incidental and untreated intracranial meningioma are relatively uncommon and are primarily single center and retrospective in design.^9^ Early studies primarily described characteristics and patterns of growth, whereas more recent studies have attempted to identify risk factors for growth.^9^ Definitions of growth or progression have not been uniform, which has hampered synthesis of results; for instance, some studies report absolute changes in tumor size, while others report relative changes in tumor size. The Response Assessment in Neuro-Oncology Meningioma Working Group has recommended that change with relation to time (rate) should be used, but this has been inconsistently applied.^14^

Recent work has attempted to accurately define risk factors for untreated meningioma growth. The **A**san **I**ntracranial **M**eningioma **S**coring **S**ystem and **I**ncidental **M**eningioma: **P**rognostic **A**nalysis Using Patient **C**omorbidity and MRI **T**ests (IMPACT) calculator stratify patients based on the imaging features of a meningioma into risk groups.^15,16^ Both scoring systems require external validation in patients with an incidental or untreated meningioma, and this could pave the way for prospective clinical studies. However, outcome measurements in studies such as these are heterogeneous, for instance, with respect to definitions of growth, and their associated metrics.

A Core Outcome Set (COS) is defined as the *minimum* set of outcomes that should be measured and reported in all clinical studies for a specific health condition or health area.^17^ COS development is in its infancy within the field of neuro-oncology, but efforts are underway.^18^ While COS are typically developed for use in randomized controlled trials, future prospective observational studies evaluating management strategies for patients with an incidental or minimally symptomatic meningioma could benefit from the implementation of a COS that is specific to this patient group. Outcomes that are considered to be core may well be different in this patient group in comparison to patients who are to receive therapeutic intervention. Harmonization of outcome measurement and reporting could reduce research waste and allow meaningful comparison of results across similar studies, in order to pool data and formulate robust treatment strategies. This will be achieved within the remit of The COSMIC Project, an international effort to develop 2 COS for meningioma. *COSMIC: Intervention* is being developed for use in phase 2 and later, intracranial meningioma clinical trials that are designed to inform clinical decision-making and improve clinical care for patients. *COSMIC: Observation* is being developed for use in observational studies concerned with incidental, minimally symptomatic, and/or untreated cohorts of patients with intracranial meningioma, that are designed to inform monitoring and decision to treatment strategies.^19^

The aim of this systematic review was to identify what outcomes have been measured and reported in observational studies of incidental and untreated intracranial meningioma and what outcomes are being measured and reported in ongoing observational clinical studies. The results of this systematic review will be used to inform a long list of outcomes of potential relevance to key stakeholders, including patients with meningioma, which will be prioritized through established consensus methodology to develop the COSMIC: Observation COS in a subsequent step.

## Research Question

What outcomes are measured and reported in ongoing and published clinical studies describing cohorts of adults with incidental and untreated intracranial meningioma?

## Methods

### Inclusion Criteria

Full-text articles reporting results of observational studies that evaluated active monitoring strategies for adult cohorts with incidental, minimally symptomatic, and/or untreated intracranial meningioma (based on a radiological diagnosis) were included. For the purposes of this systematic review, active monitoring was considered to be an intervention and included clinical review (including history and clinical examination), testing (for instance, to obtain patient-reported, caregiver-reported, or performance outcomes), and imaging (using any modality and with any frequency). A minimum of 20 intracranial meningioma patients per study was required. Patients were adults (18 years and above) of either sex, with a radiological diagnosis of sporadic intracranial meningioma, including patients with multiple meningioma and SMARCE1 loss-related familial meningioma. Multiple publications relating to the same study were included but considered together, so repetition of data extraction was not performed (for instance, interim results and subgroup analyses). Studies with a mix of brain tumor types whereby at least 20 patients had an intracranial meningioma were included. Online international trial registries were searched to identify ongoing trials meeting the aforementioned criteria (with an expected accrual greater than 20 patients). Only published trials and online trial registry entries written in the English language were included, due to limitations on resources.

### Exclusion Criteria

Studies were excluded if they included fewer than 20 patients or if they principally described cohorts with spinal meningioma, radiation-induced meningioma (eg, administered in childhood as an intervention for cancer), or associated with the genetic condition NF2-schwannomatosis.

### Information Sources and Search Strategy

A detailed search strategy utilizing the search strings “meningioma” AND “incidental” OR “untreated” was developed and translated to interrogate the following electronic bibliographic databases: PubMed, EMBASE, MEDLINE, and CINAHL via EBSCO. In addition, simple searches of the following trial registries were conducted: Cochrane Central Register of Controlled Trials, ClinicalTrials.gov, and the WHO International Clinical Trials Registry Platform. The search strategies are provided in Supplementary Appendix 1. The searches were first run on June 2, 2021. The searches were re-run on January 24, 2022, to identify any new records published since the first search.

### Selection Process

Search results were downloaded from their respective online databases, and uploaded to the online platform Rayyan.^20^ Following de-duplication, 2 review authors (C.P.M. and A.I.I.) independently screened all titles and abstracts that were retrieved according to the eligibility criteria. Screening was performed on the Rayyan platform independently, and each review author was blind to the screening choices made by the other review author. For titles and abstracts that appeared to meet the eligibility criteria, and for those where a decision could not be confidently made based on title and abstract alone, full-text copies were obtained. All full-text copies were independently screened to assess for eligibility by the same 2 review authors (C.P.M. and A.I.I.). No full-text eligibility checks required escalation to the senior review author (M.D.J.). The complete reference list of full-text titles included was screened to identify titles not identified through the searches. Trial registry searches were independently performed by a single review author and screened against the same eligibility criteria (CPM) to identify ongoing studies not yet published that describe outcomes that will be measured and reported.

### Data Items and Data Collection Process

Data were extracted from eligible articles and trial registry entries by a single review author (C.P.M.) into a custom-designed and piloted spreadsheet in Microsoft Excel (v16.34, Microsoft) following best practice described by the COMET Initiative.^17,21^ The first 10% of included titles were dual extracted by a second review author and confirmed consistency and accuracy of extraction (A.I.I.).

The following data were extracted from each study as recommended by COMET^17,21^: study type, study population, first author, year and journal of publication, intervention(s) under investigation, each outcome reported (recorded verbatim) from the study abstract, methods, or results, the definition of the outcome if provided, and whether it was a primary or secondary outcome if stated. The indicator and/or tool(s) used to operationalize or measure the outcome were also extracted when available. The number of verbatim outcomes per trial/study was recorded.

A trial or study outcome is a measurable variable examined in response to a treatment or intervention (including active monitoring for the purpose of this study). An outcome was defined as “one that has original meaning and context.”^22^ Identical outcomes measured at multiple time points were not extracted as different unique outcomes.

### Synthesis Methods

Tabulation and descriptive data analysis were performed in Microsoft Excel (v16.34, Microsoft) with the aim of de-duplicating verbatim outcomes extracted from included studies into a list of unique outcomes, followed by grouping of unique outcomes under standardized outcome terms where similar meaning and context exists. Given that there exists considerable heterogeneity in the definition of what constitutes a unique outcome, we utilized the method of data analysis as per Young et al.^22^ and classified outcomes according to the outcome framework proposed by COMET.^17,23^

### Registration and Protocol

This study is registered with the Core Outcome Measures in Effectiveness Trials (COMET) database as study 1508 and accessible at (https://www.comet-initiative.org/Studies/Details/1508). Institutional review board (University of Liverpool) sponsorship and ethical approval have been obtained for The COSMIC Project (Ref UoL001601).

The review question and question format are summarized in Table 1.

**Table 1.** SDMO (Studies, Data, Methods, and Outcomes) Table Summarizing Review Question and Question Format Structure

Review question	What outcomes are measured and reported in ongoing and published observational studies describing cohorts of adults with incidental and untreated intracranial meningioma?
Types of studies	Published or ongoing observational studies reporting results of active monitoring. Minimum of 20 patients recruited or planned.
Types of data	Trial outcomes reported by article and registry authors that have been measured or plan to be measured in actively monitored cohorts.
Types of methods	Choice of outcomes to be measured including outcome definition, method of measurement, and time-point of measurement.
Outcomes	Heterogeneity of outcome measurement and reporting across clinical studies.

## Results

### Studies Identified

**Figure 1.**
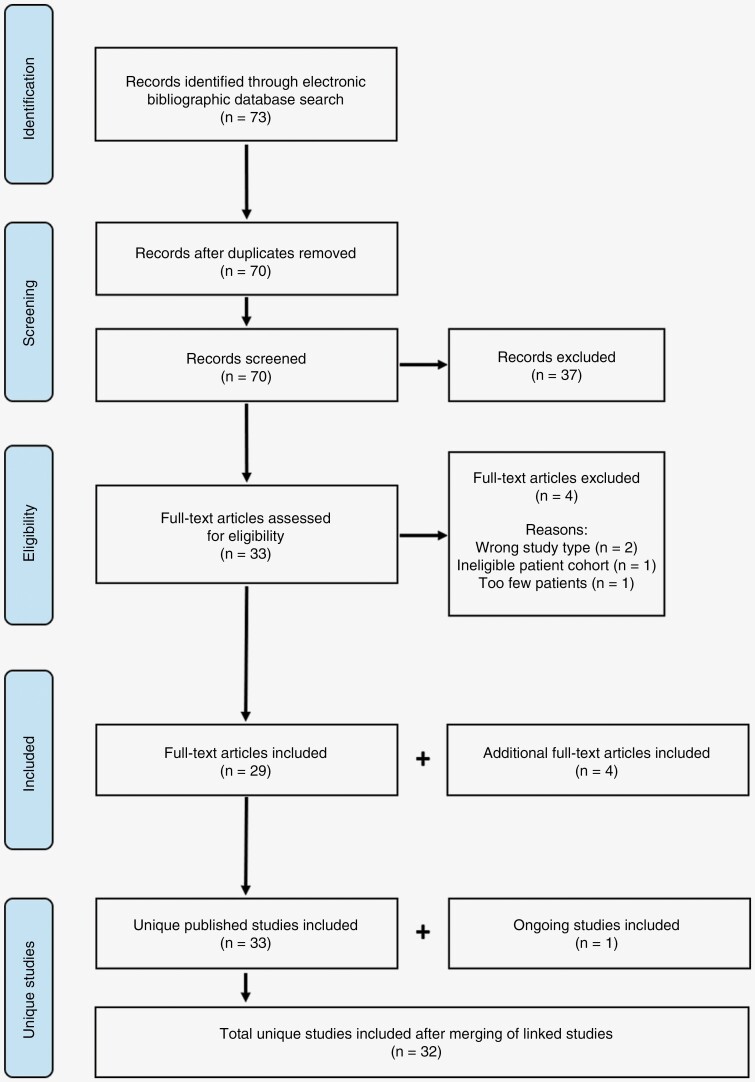
PRISMA flow diagram depicting the identification, screening, eligibility, and inclusion of unique published studies and ongoing studies.

From 73 records identified following electronic bibliographic database searching, 70 were screened for inclusion after duplicates were removed, and 33 remained for full-text article eligibility checks. Four full-text articles were excluded due to the wrong study type (*n* = 2), ineligible patient cohort (*n* = 1), and too few patients (*n* = 1). Four additional full-text articles were identified and included following hand-searching of the literature, and 1 ongoing study was identified and included. After merging of linked full-texts, 32 unique studies were identified and included in the systematic review (Figure 1). Table 2 shows a summary of the characteristics of the 32 studies (details of the full texts are in Supplementary Appendix 2).

**Table 2.** Summary of Characteristics of Studies Included in Systematic Review

Characteristic		*N* (No. of Studies)
Number of unique studies identified		32
Year of publication	1990–1999	2
	2000–2009	9
	2010–2019	13
	2020–2022	8
Study type	Retrospective case-series	21
	Retrospective cohort study	5
	Retrospective case–control study	1
	Prospective cross-sectional study	3
	Prospective cohort study	2

		*N* (No. of patients)
Study population	Total no. in systematic review	4177
	Median no. per study	67

		*N* (No. of outcomes)
Study outcomes	Extracted	267
	Median no. per study	7.5
	With primary outcome designation	17
	With secondary outcome designation	17
	Defined	87

### Outcomes Reported

In total, 267 individual verbatim outcome terms were identified from the 32 included studies. Following the de-duplication of identical outcomes (including those with variation in spelling, eg, tumour and tumor), 178 unique verbatim outcome terms remained. A standardized outcome term was selected and applied to each unique verbatim outcome term in order to group those with similar meaning, for example, “absolute annual growth rate” and “annual growth rate” were grouped under “absolute growth rate”. Two additional review authors checked the appropriateness and consistency of the standardized outcome terms applied to the unique verbatim outcome terms (A.I.I. and M.D.J.). This resulted in 53 standardized outcome terms. The unique verbatim outcome terms, their frequency of reporting, and the applied standardized outcome terms are listed in Supplementary Appendix 3. The final list of standardized outcome terms, their reporting frequency, and the number of those defined within their study of origin are listed in Supplementary Appendix 4.

### Outcome Definitions

**Figure 2.**
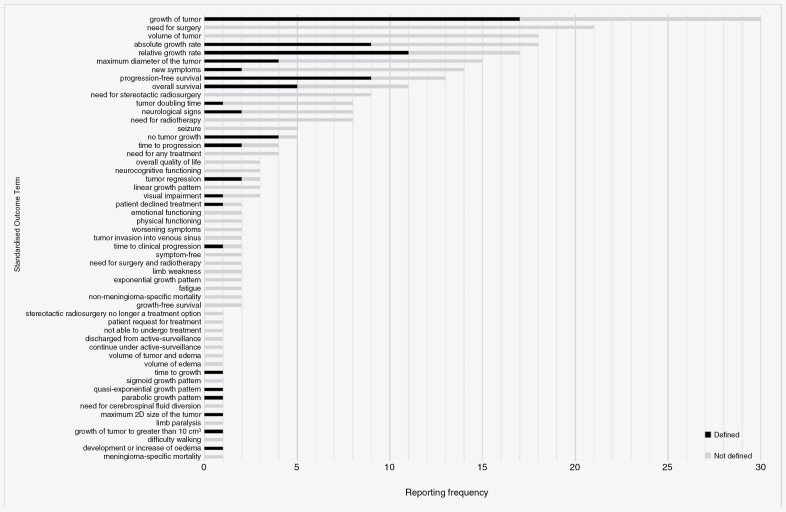
Reporting frequency of standardized outcome terms, along with the proportion of those defined vs undefined for each.

Of the 267 individual verbatim outcome terms identified in the included studies, 77 (29%) were accompanied by an outcome definition. Figure 2 shows the reporting frequency of each standardized outcome term and the proportion of each defined and undefined.

### Mapping of Standardized Outcome Terms to the COMET Taxonomy

Each standardized outcome term was mapped to a COMET outcome domain. In total, 9 domains are represented. The 9 domains map to 3 overarching COMET core areas namely, death, physiological/clinical, and life impact (Supplementary Appendix 4). Table 3 shows the number of studies reporting an individual outcome from each outcome domain, the number of unique outcomes from each domain, and the number of standardized outcome terms from each domain.

**Table 3.** COMET Outcome Domains and their COMET core areas identified in the systematic review

COMET Core Area	COMET Outcome Domain and No.	Studies	Individual Outcomes	Unique Outcomes	Standardized Outcome Terms
Death	Mortality/survival (1)	10	29	15	5
Physiological/clinical	Eye outcomes (7)	4	4	3	1
	General outcomes (9)	2	2	1	1
	Nervous system outcomes (17)	31	215	142	36
Life impact	Physical functioning (25)	2	2	2	1
	Emotional functioning/wellbeing (28)	2	2	2	1
	Cognitive functioning (29)	3	3	3	1
	Global Quality of Life (30)	3	3	3	1
	Delivery of care (32)	3	7	7	6
Total	9	32	267	178	53

### COMET Core Areas and COMET Outcome Domains Represented

#### COMET core area “Death”.—

Five standardized outcome terms mapped to the COMET outcome domain “mortality/survival” and 10 studies (31%) reported an outcome from this domain. Three of these standardized outcome terms concern binary events, namely “meningioma-specific mortality,” “non-meningioma-specific mortality,” and “overall survival,” while 2 were composite outcomes, “growth-free survival” and “progression-free survival” (Supplementary Appendix 5). Heterogeneous outcome definitions were identified for “overall survival” (total definitions *n* = 5, unique definitions *n* = 4) and “progression-free survival” (total definitions *n* = 9, unique definitions *n* = 8).

#### COMET core area “Physiological/clinical”.—

Thirty-six standardized outcome terms mapped to the COMET outcome domain “nervous system outcomes” and 31 studies (97%) reported an outcome from this domain. One standardized outcome term mapped to “eye outcomes” and was reported by 4 studies (13%), and another standardized outcome term mapped to “general outcomes” and was reported by 2 studies (6%).

When considered together, several distinct groups of standardized outcome terms emerged (see Supplementary Appendix 5). Four time-to-event standardized outcome terms addressed growth and progression. Two binary events standardized outcome terms addressed clinical/radiological stability. Two binary events standardized outcome terms addressing growth that precludes specific modalities of treatment. Six binary events standardized outcome terms concerning the need for treatments. Five clinician reported multiple category event standardized outcome terms addressing size and volume of tumor and edema. Ten clinician reported multiple category event standardized outcome terms addressed tumor growth, growth rate, and growth pattern. A clinician reported multiple category event standardized outcome terms addressing the development of neurological signs. Eight patient reported multiple category event standardized outcome terms addressing symptom development.

Within this COMET core area, infrequent and heterogeneous outcome definitions were identified for a number of frequently reported standardized outcome terms which included “maximum diameter of the tumor” (total definitions *n* = 4, unique definitions *n* = 4), “growth of tumor” (total definitions *n* = 17, unique definitions *n* = 17), “absolute growth rate” (total definitions *n* = 9, unique definitions *n* = 7), “relative growth rate” (total definitions *n* = 11, unique definitions *n* = 10), and “new symptoms” (total definitions *n* = 2, unique definitions *n* = 2). In addition, 2 frequently reported standardized outcome terms were never defined within studies, including “need for surgery” and “tumor volume.”

#### COMET core area “Life impact”.—

One standardized outcome term mapped to each of the functioning domains of “physical functioning,” “emotional functioning/wellbeing” and “cognitive functioning,” and 2 studies (6%), 2 studies (6%), and 3 studies (9%) reported an outcome from each of these domains respectively. All 3 of these standardized outcome terms are multidimensional health measures, none of which were defined (Supplementary Appendix 5). One standardized outcome term mapped to the COMET outcome domain “global quality of life” and 3 studies (9%) reported an outcome from this domain. This standardized outcome term is also a multidimensional health measure and was not defined (Supplementary Appendix 5). Six standardized outcome terms mapped to the COMET outcome domain “delivery of care” and 3 studies (9%) reported an outcome from this domain. All 6 of these standardized outcome terms are binary events, but only 1 definition was identified which was for the standardized outcome term “patient declined treatment” (Supplementary Appendix 5).

### Indicator and/or Tool(s) Used to Operationalize or Measure Standardized Outcome Terms

Outcomes must be operationalized or measured and where possible, we have extracted the indicator and/or tool used to do this, as reported in the included studies. This information will be used after the COSMIC: Observation COS has been defined, in order to begin the process of selecting indicators and/or tools for operationalizing or measuring each included core outcome.

For binary event and time-to-event standardized outcome terms, definitions of the events must be established. Similarly, for composite outcome standardized outcome terms, definitions of events must be established but also the events included. For clinician-reported multiple category event standardized outcome terms, definitions of the events must again be established but also an indicator and/or tool(s). Finally, for multidimensional health measures, a tool(s) must be selected. We have extracted existing definitions from studies as previously described, but also extracted indicators and/or tools where available.

#### Indicator and/or tool(s) used to operationalize or measure multiple category event outcomes.—

All 16 clinician reported multiple category event standardized outcome terms identified had at least 1 associated indicator and/or tool(s) identified (summarized in Supplementary Appendix 6). Four were identified including radiological measurement (specified as being performed with MRI, CT, or MRI, or not specified), radiological assessment as per RANO criteria, mathematical calculations (performed from existing or acquired data points), and the Neurologic Functional Status Scale, used specifically in 1 study evaluating the standardized outcome term “neurological signs.” For the purpose of this work, a comprehensive analysis of heterogeneity is not yet required, as which standardized outcome terms will be included in the COSMIC: Observation COS is yet to be determined.

Of the 8 patient reported multiple category event standardized outcome terms identified, 6 concerned specific symptoms, while 2 concerned symptoms as a whole, “worsening symptoms” and “new symptoms” (summarized in Supplementary Appendix 6). The only tools identified were those associated with the standardized outcome term “fatigue,” which included “Profile of Mood States” and “Brief Fatigue Inventory” and the Brain Cancer Module (BCM20) associated with the standardized outcome term “new symptoms.”

#### Indicator and/or tool(s) used to operationalize or measure multidimensional health measures.—

At least 1 instrument was associated with each of the four multidimensional health measure standardized outcome terms identified (summarized in Supplementary Appendix 6). The Barthel Index and Karnofsky Performance Status scale were associated with “Physical functioning,” the Hospital Anxiety and Depression Scale was associated with “emotional functioning,” 2 neurocognitive test batteries were associated with “neurocognitive functioning,” and the 36-Item Short Form Survey (SF-36) and the EORTC Core Quality of Life questionnaire (EORTC QLQ-C30) were associated with “overall quality of life” (only items 29 and 30 were utilized).

## Discussion

Through a systematic approach, we have identified 178 unique outcomes measured and reported in 32 clinical studies of incidental, minimally symptomatic, and untreated intracranial meningioma. After grouping unique outcomes with the same or similar meaning, we generated 53 standardized outcome terms which were classified using the COMET taxonomy into 9 outcome domains and 3 core areas. Two-thirds of the standardized outcome terms generated were from the domain “nervous system outcomes.” The most frequently reported standardized outcome terms were “maximum diameter of the tumor,” “growth of tumor,” “absolute growth rate,” “relative growth rate,” and “new symptoms”. However, outcome definitions and methods of outcome measurement were heterogeneous and not always reported. A third of the studies included a “mortality/survival” outcome.

Healthcare professionals and patients want to know if an asymptomatic intracranial meningioma will grow and become symptomatic, such that it will require treatment within their lifetime. However, the absence of high-quality observational evidence to inform clinical practice probably results in patients undergoing unnecessary interventions, having prolonged periods of observation, or even loss of therapeutic options or curability. A quarter of the studies included in this systematic review were published after the year 2020. This demonstrates the increasing interest in this health area by healthcare professionals and researchers, which may be a response to greater detection of incidental intracranial meningioma detected with the increased use of MRI. However, the majority of these observational studies are retrospective, with large limitations in study design. This likely adds to the heterogeneity in outcome measurement observed and further justifies the development of a COS for this health condition.

This is the first systematic review identifying outcomes measured and reported in patients with incidental, minimally symptomatic, and untreated intracranial meningioma. We have applied rigorous methodology to identify studies and their outcomes, followed by domain categorization with study advisory input from experts. The 3 “functioning” domains and the domain “global quality of life” were reported infrequently. We know that patients with asymptomatic, incidental, and untreated intracranial meningioma suffer from a wide range of issues, including impaired functioning and health-related quality of life.^24^ Therefore, although we identified these domains infrequently, they will be advanced through consensus methodology to allow key stakeholders to rate and discuss their importance for inclusion in the COSMIC: Observation COS.

There are some limitations to this systematic review. First, the searches identified full texts written only in the English language. Therefore, there may be studies and, moreover, unique outcomes that we have not identified. In addition, only full-texts with a minimum of 20 patients were included. This arbitrary cut-off served to exclude studies with low patient numbers, that are likely to be of lower quality and limited value to the review process. One does not need to identify every paper of relevance, as the purpose of this methodological review is to sample the literature. This is an accepted method for limiting the search results when the objective is to perform a methodological sampling review. Despite both of these limitations, we generated 53 standardized outcome terms, and are confident that an accurate breadth has been achieved. Furthermore, participants recruited to the latter stages of The COSMIC Project will have the opportunity to add new outcomes that they feel are not represented by those in the eDelphi survey, and this should mitigate against the loss of unique outcomes that we may have missed. Data extraction was performed by a single review author; the principal investigator for The COSMIC Project (CPM). While dual extraction of data is preferable, there were both financial and personnel limitations preventing this. This was mitigated by maintaining a low threshold for extracting potential unique study outcomes to ensure none were missed. As the first 10% of included studies were dual extracted by a second review author (A.I.I.), we believe consistency and accuracy of extraction was achieved.

The purpose of this methodological review was to enable the development of a COS for observational studies of patients with incidental and untreated meningioma. Therefore, it is important that the types of studies included in this review contribute data that is both applicable and relevant to the target population that shall be the subject of the COS that is developed. To this end, it was necessary to exclude studies that included patients with NF2-schwannomatosis as these studies would invariably include outcomes that are reflective of the wider syndrome, thereby confounding the COS development process. This strategy was strongly supported by the study advisory group. The best practice would be to develop a COS that is specific to NF2-schwannomatosis, which utilizes the NF2-schwannomatosis literature and includes representative stakeholders, including patients with NF2-schwannomatosis to develop a COS that includes outcomes that are most relevant to this unique patient population. Conversely, there was no good scientific reason to exclude SMARCE1 loss-related familial meningioma or cohorts of patients with multiple meningioma as no confounding factors could be identified that would have hampered the COS development process. Therefore, these nuanced examples of patient populations that were eligible for inclusion have been exemplifed for transparency. Ultimately, the eligibility criteria for inclusion of studies in this methodological review (and the scope of the COS that is ultimately developed), cannot be too broad, or inclusive of patient populations that would confound the core outcomes advanced to the final COS (which may not be relevant to all patients with an incidental or untreated intracranial meningioma undergoing observation).

There is increasing interest in the natural history and clinical management of patients with incidental intracranial meningioma, and future prospective studies are anticipated. Our findings in this review demonstrate that the outcomes measured and reported in the relevant literature are both heterogeneous as well as poorly and variably defined. Development of a COS for future clinical studies of incidental and untreated intracranial meningioma is therefore justified and will harmonize outcome reporting and reduce research waste for this health area. The standardized outcome terms generated in this systematic review will be rationalized and used to populate a modified eDelphi survey which will be completed by key stakeholders, including patients. A consensus meeting of key stakeholders will take place to ratify the final COSMIC: Observation COS. This process of information gathering followed by consensus methodology follows best practices as outlined by COMET. Further work will be required to determine how to measure each core outcome, but data generated from this systematic review on “how” outcomes were measured will provide the basis for this.

Collaborators

International Consortium on Meningioma: Kenneth Aldape, Abdurrahman I. Islim, Karolyn Au, Jill Barnhartz-Sloan, Wenya Linda Bi, Felix Behling, Priscilla K. Brastianos, Chaya Brodie, Nicholas Butowski, Carlos Carlotti, Ana Castro, Aaron Cohen-Gadol, Marta Couce, Michael D. Cusimano, Francesco DiMeco, Katharine Drummond, Ian F. Dunn, Craig Erker, Michelle Felicella, Daniel M. Fountain, Evanthia Galanis, Norbert Galldiks, Caterina Giannini, Roland Goldbrunner, Brent Griffith, Rintaro Hashizume, C. Oliver Hanemann, Christel Herold-Mende, Luke Hnenny, Craig Horbinski, Raymond Y. Huang, David James, Michael D. Jenkinson, Christine Jungk, Gerhard Jungwirth, Timothy J. Kaufmann, Boris Krischek, Sylvia Kurz, Daniel Lachance, Christian Lafougère, Katrin Lamszus, Ian Lee, Jeff C. Liu, Serge Makarenko, Tathiana Malta, Yasin Mamatjan, Alireza Mansouri, Christian Mawrin, Michael McDermott, Christopher P. Millward, Jennifer Moliterno-Gunel, Andrew Morokoff, David Munoz, Farshad Nassiri, Houtan Noushmehr, Ho-Keung Ng, Arie Perry, Farhad Pirouzmand, Laila M Poisson, Bianca Pollo, Aditya Ragunathan, David R. Raleigh, Mirjam Renovanz, Franz Ricklefs, Felix Sahm, Andrea Saladino, Antonio Santacroce, Thomas Santarius, Jens Schittenhelm, Christian Schichor, David Schultz, Nils O. Schmidt, Warren Selman, Helen Shih, Andrew Sloan, Julian Spears, Matija Snuderl, James Snyder, Suganth Suppiah, Erik Sulman, Ghazaleh Tabatabai, Marcos Tatagiba, Marco Timmer, Daniela Tirapelli, Joerg C. Tonn, Derek Tsang, Michael A. Vogelbaum, Andreas von Deimling, Tobias Walbert, Simon Walling, Justin Z. Wang, Patrick Y. Wen, Manfred Westphal, Adriana M. Workewych, Stephen Yip, Gabriel Zada, Gelareh Zadeh, Viktor Zherebitskiy.

## Supplementary Material

vdae042_suppl_Supplementary_Appendix
