# Supplementary material for: The outcomes measured and reported in observational studies of incidental and untreated intracranial meningioma: A systematic review
Source: Neurooncol Adv. 2024 Mar 19;6(1):vdae042. doi: 10.1093/noajnl/vdae042 (PMC11003528; doi:10.1093/noajnl/vdae042)
Supplement: vdae042_suppl_Supplementary_Appendix [file vdae042_suppl_supplementary_appendix.docx]

**Supplementary Appendix 1 - Database and Trial Registry Searches**

**Medline (Ovid)**

| Search | Query |
| --- | --- |
| 1 | exp meningioma/ |
| 2 | ((central nervous system or CNS or brain* or cerebral* or intracranial or intra-cranial) adj3 (cancer* or tumo?r* or malignan* or neoplas*)).mp. |
| 3 | 1 or 2 |
| 4 | (glioma* or glial* or glioblastoma* or GBM* or astrocytoma* or ependym* or subependym* or neurocytoma* or pineal* or pineo* or chordoma* or hamartoma* or pituitary* or craniopharyngioma* or neuroblastoma* or medulloblastoma* or lymphoma* or metastat*).mp. |
| 5 | ((lung* or breast* or skin* or blood* or h?ematolg* or dermatolog* or renal* or genitourinary*) adj3 (cancer* or tumo?r* or malignan* or neoplas*)).mp. |
| 6 | (leuk?emia* or myeloma* melanoma*).mp. |
| 7 | 4 or 5 or 6 |
| 8 | 3 not 7 |
| 9 | (asymptomatic or incidental or small or untreated).mp. |
| 10 | (surgery or radiotherapy or radiosurg* or observ* or conservative treatment or follow-up or natural history or growth).mp. |
| 11 | 8 and 9 and 10 |
| 12 | Limit 11 to English |

**EMBASE (Ovid)**

| Search | Query |
| --- | --- |
| 1 | exp meningioma/ |
| 2 | ((central nervous system or CNS or brain* or cerebral* or intracranial or intra-cranial) adj3 (cancer* or tumo?r* or malignan* or neoplas*)).mp. |
| 3 | 1 or 2 |
| 4 | (glioma* or glial* or glioblastoma* or GBM* or astrocytoma* or ependym* or subependym* or neurocytoma* or pineal* or pineo* or chordoma* or hamartoma* or pituitary* or craniopharyngioma* or neuroblastoma* or medulloblastoma* or lymphoma* or metastat*).mp. |
| 5 | ((lung* or breast* or skin* or blood* or h?ematolg* or dermatolog* or renal* or genitourinary*) adj3 (cancer* or tumo?r* or malignan* or neoplas*)).mp. |
| 6 | (leuk?emia* or myeloma* melanoma*).mp. |
| 7 | 4 or 5 or 6 |
| 8 | 3 not 7 |
| 9 | (asymptomatic or incidental or small or untreated).mp. |
| 10 | (surgery or radiotherapy or radiosurg* or observ* or conservative treatment or follow-up or natural history or growth).mp. |
| 11 | 8 and 9 and 10 |
| 12 | Limit 11 to English |

**CINAHL Plus**

| Search | Query |
| --- | --- |
| 1 | Meningioma |
| 2 | ((central nervous system or CNS or brain* or cerebral* or intracranial or intra-cranial) N3 (cancer* or tumo?r* or malignan* or neoplas*)) |
| 3 | 1 or 2 |
| 4 | (glioma* or glial* or glioblastoma* or GBM* or astrocytoma* or ependym* or subependym* or neurocytoma* or pineal* or pineo* or chordoma* or hamartoma* or pituitary* or craniopharyngioma* or neuroblastoma* or medulloblastoma* or lymphoma* or metastat*) |
| 5 | ((lung* or breast* or skin* or blood* or h?ematolg* or dermatolog* or renal* or genitourinary*) N3 (cancer* or tumo?r* or malignan* or neoplas*)) |
| 6 | (leuk?emia* or myeloma* melanoma*) |
| 7 | 4 or 5 or 6 |
| 8 | 3 not 7 |
| 9 | (asymptomatic or incidental or small or untreated) |
| 10 | (surgery or radiotherapy or radiosurg* or observ* or conservative treatment or follow-up or natural history or growth) |
| 11 | 8 and 9 and 10 |
| 12 | Limit 11 to English |

**Cochrane central register of controlled trials**

| Search | Query |
| --- | --- |
| 1 | Meningioma |
| 2 | ((central nervous system or CNS or brain* or cerebral* or intracranial or intra-cranial) NEAR/3 (cancer* or tumo?r* or malignan* or neoplas*)) |
| 3 | 1 or 2 |
| 4 | (glioma* or glial* or glioblastoma* or GBM* or astrocytoma* or ependym* or subependym* or neurocytoma* or pineal* or pineo* or chordoma* or hamartoma* or pituitary* or craniopharyngioma* or neuroblastoma* or medulloblastoma* or lymphoma* or metastat*) |
| 5 | ((lung* or breast* or skin* or blood* or h?ematolg* or dermatolog* or renal* or genitourinary*) NEAR/3 (cancer* or tumo?r* or malignan* or neoplas*)) |
| 6 | (leuk?emia* or myeloma* melanoma*) |
| 7 | 4 or 5 or 6 |
| 8 | 3 not 7 |
| 9 | (asymptomatic or incidental or small or untreated) |
| 10 | (surgery or radiotherapy or radiosurg* or observ* or conservative treatment or follow-up or natural history or growth) |
| 11 | 8 and 9 and 10 |
| 12 | Limit 11 to English |

**ClinicalTrials.gov**

Search Condition or disease – meningioma

Filters (include) – Recruitment (not yet recruiting, recruiting, enrolling by invitation, active not recruiting, suspended, completed)

Filters (include) – Study type (observational)

**WHO International Clinical Trials Registry Platform**

Search – meningioma

Filters - None

**Supplementary Appendix 2 – Studies Included in Systematic Review**

| **ID** | **Author** | **Study Title** | **Study Type** | **Study Population** | **Interventions** | **Outcomes**  **(No. / study)** |
| --- | --- | --- | --- | --- | --- | --- |
| 1 | Olivero et al. (1995) | The natural history and growth rate of asymptomatic meningiomas: A review of 60 patients | Retrospective  case-series | Asymptomatic patients’  Not wanting surgery  (n=60) | Clinical & radiological F/U | 4 |
| 2 | Go et al. (1998) | The natural history of asymptomatic meningiomas in Olmsted County, Minnesota | Retrospective  case-series | Asymptomatic patients’ Incidental meningioma  (n=35) | Clinical & radiological F/U | 8 |
| 3 | Kuratsu et al. (2000) | Incidence and clinical features of asymptomatic meningiomas | Retrospective  case-series | Asymptomatic patients’  (n=196) | Clinical & radiological F/U | 3 |
| 4 | Niiro et al. (2000) | Natural history of elderly patients with asymptomatic meningiomas | Retrospective  case-series | Asymptomatic patients’  Elderly (over 70)  (n=40) | Clinical & radiological F/U | 6 |
| 5 | Yoneoka et al (2000) | Growth of incidental meningiomas | Retrospective  case-series | Asymptomatic patients’  Incidental meningioma  CT and/or MRI F/U > 6/12  (n=37) | Clinical & radiological F/U | 2 |
| 6 | Bindal et al. (2003) | The natural history of untreated skull base meningiomas | Retrospective  case-series | Skull base meningioma  Untreated or long delay  (n=40) | Clinical & radiological F/U | 6 |
| 7 | Nakamura et al. (2003) | The natural history of incidental meningiomas | Retrospective  case-series | Asymptomatic patients’  Under F/U  (n=47) | Clinical & radiological F/U | 5 |
| 8 | Herscovici et al. (2004) | Natural history of conservatively treated meningiomas | Retrospective  case-series | Asymptomatic patients’ Incidental meningioma  Under F/U  (n=43) | Clinical & radiological F/U | 5 |
| 9 | Yano et al. (2006) | Indications for surgery in patients with asymptomatic meningiomas based on an extensive experience | Retrospective  cohort study | Asymptomatic patients  Under F/U  (n=603) | Clinical & radiological F/U | 11 |
| 10 | Zeidman et al. (2008) | Growth rate of non-operated meningiomas | Retrospective  case-series | Non-operated meningioma  (n=21) | Clinical & radiological F/U | 4 |
| 11 | Hashiba et al. (2009) | Serial volumetric assessment of the natural history and growth pattern of incidentally discovered meningiomas | Retrospective  case-series | Asymptomatic patients’ Incidental meningioma  3x MRI over at least 1 year  (n=70) | Clinical & radiological F/U | 9 |
| 12 | Jo et al. (2011) | Treatment modalities and outcomes for asymptomatic meningiomas | Retrospective  case-series | Asymptomatic patients’  (n=146) | Clinical & radiological F/U | 8 |
| 13 | Oya et al. (2011) | The natural history of intracranial meningiomas: Clinical article | Retrospective  case-series | Conservatively managed patients with at least 1 year F/U  (n=238) | Clinical & radiological F/U | 8 |
| 14 | Rubin et al. (2011) | Outcome of untreated meningiomas | Retrospective  case-series | Conservatively treated patients’  Mostly incidental meningioma  (n=56) | Clinical & radiological F/U | 3 |
| 15 | Hashimoto et al. (2012) | Slower growth of skull base meningiomas compared with non-skull base meningiomas based on volumetric and biological studies: Clinical article | Retrospective  case-series | Incidental meningioma  3x MRI over at least 1 year  (n=110) | Clinical & radiological F/U | 11 |
| 16 | Van Nieuwenhuizen et al. (2013) | Neurocognitive functioning and health-related quality of life in patients with radiologically suspected meningiomas | Prospective cross-sectional study | Untreated patients with stable disease, under watch-and-wait  (n=21) | Assessment of neurocognitive functioning, HRQOL, neurologic functioning, and performance status | 8 |
| 17 | Jadid et al. (2015) | Long-term follow-up of incidentally discovered meningiomas | Retrospective  case-series | Asymptomatic patients’ Incidental meningioma  (n=65) | Clinical & radiological F/U | 5 |
| 18 | Hunter et al. (2017) | The Natural History of Petroclival Meningiomas: A Volumetric Study | Retrospective  case-series | Petroclival meningioma  Untreated patients’, 2x MRI separated by min 3 months  (n=34) | Clinical & radiological F/U | 8 |
| 19 | Lee et al. (2017) | A novel weighted scoring system for estimating the risk of rapid growth in untreated intracranial meningiomas | Retrospective  case-series | Incidental meningioma  Under F/U  (n=232) | Clinical & radiological F/U | 11 |
| 20 | Lee et al. (2017) | "Wait-and-see" strategies for newly diagnosed intracranial meningiomas based on the risk of future observation failure | Retrospective  case-series | Untreated patients’  Under 'wait-and-see'  (n=232) | Clinical & radiological F/U | 16 |
| 21 | Goebel et al. (2018) | A missing piece? Neuropsychiatric functioning in untreated patients with tumors within the cerebellopontine angle | Prospective cross-sectional study | Patients with untreated CPA tumours Inc. meningioma  (n=54) | Assessment of cognition, mood, and fatigue | 4 |
| 22 | Romani et al. (2018) | Non-operative meningiomas: long-term follow-up of 136 patients | Retrospective  case-series | Incidental meningioma  Under F/U  (n=136) | Clinical & radiological F/U | 8 |
| 23 | Behbahani et al. (2019) | A prospective study of the natural history of incidental meningioma-Hold your horses! | Prospective cohort study | Incidental meningioma  Under F/U for 5 years  (n=64) | Clinical & radiological F/U | 15 |
| 24 | Brugada-Bellsola et al. (2019) | Growth prediction in asymptomatic meningiomas: the utility of the AIMSS score | Retrospective  case-series | Asymptomatic patients  (n=69) | Clinical & radiological F/U | 7 |
| 25 | Dresser et al. (2020) | Estrogen hormone replacement therapy in incidental intracranial meningioma: a growth-rate analysis | Retrospective  case-controlled study | Incidental meningioma  Received estrogen HRT or not  (n=120) | Clinical & radiological F/U | 7 |
| 26a | Islim et al. (2020) | A prognostic model to personalize monitoring regimes for patients with incidental asymptomatic meningiomas | Retrospective  case-series | Asymptomatic patients’ Incidental meningioma  (n=385) | Clinical & radiological F/U | 20 |
| 26b | Islim et al. (2022) | External validation and recalibration of an incidental meningioma prognostic model – IMPACT: protocol for an international multicentre retrospective cohort study | Retrospective cohort study  (Ongoing) | Asymptomatic patients’  Incidental meningioma | Clinical & radiological F/U | 0 |
| 27 | Kalasauskas et al. (2020) | Psychological Burden in Meningioma Patients under a Wait-and-Watch Strategy and after Complete Resection Is High - Results of a Prospective Single Center Study | Prospective cross-sectional study | Asymptomatic patients  Under ‘wait-and-watch’  (n=62) | Assessment of psychological burden, HRQoL, and fatigue | 4 |
| 28 | Delago-López et al. (2021) | Volumetric growth rate of incidental asymptomatic meningiomas: a single-center prospective cohort study | Prospective cohort study | Asymptomatic patients’  Incidental meningioma  (n=85) | Clinical & radiological F/U | 19 |
| 29 | Benjamin et al. (2021) | Volumetric growth rates of untreated cavernous sinus meningiomas | Retrospective  case-series | Mostly asymptomatic patients Untreated cavernous sinus meningioma  (n=37) | Clinical & radiological F/U | 15 |
| 30 | Yamada et al. (2021) | The Impact of 5-Year Tumor Doubling Time to Predict the Subsequent Long-Term Natural History of Asymptomatic Meningiomas | Retrospective  cohort study | Asymptomatic patients’  Untreated for 5 years  (n=201) | Clinical & radiological F/U | 6 |
| 31 | Sheehan et al. (2021) | An international multicenter matched cohort analysis of incidental meningioma progression during active surveillance or after stereotactic radiosurgery: the IMPASSE study | Retrospective  cohort study | Asymptomatic patients  (n=388) | Clinical & radiological F/U | 6 |
| 32a | Mantziaris et al. (2022) | Stereotactic radiosurgery versus active surveillance for asymptomatic, skull-based meningiomas: an international, multicenter matched cohort study | Retrospective  cohort study | Asymptomatic patients’  Skull-based meningioma  (n=110) | Clinical & radiological F/U | 15 |
| 32b | Mantziaris et al. (2022) | Stereotactic radiosurgery versus active surveillance for incidental, convexity meningiomas: a matched cohort analysis from the IMPASSE study | Retrospective  cohort study | Asymptomatic patients’  Convexity meningioma  (n=140) | Clinical & radiological F/U | 0 |

**Supplementary Appendix 3 - Standardised Outcome Terms applied to Unique Verbatim Outcomes**

| **Unique verbatim outcome term** | **Reporting frequency** | **Standardised outcome term applied** |
| --- | --- | --- |
| absolute annual growth rate (AGR) | 1 | absolute growth rate |
| absolute growth rate | 6 | absolute growth rate |
| annual growth rate | 4 | absolute growth rate |
| growth rate | 1 | absolute growth rate |
| growth rate in cm3 per year | 1 | absolute growth rate |
| overall tumor growth rate | 1 | absolute growth rate |
| rapid-growth | 1 | absolute growth rate |
| tumor growth rate | 1 | absolute growth rate |
| tumor volume increase | 1 | absolute growth rate |
| volumetric growth rate | 1 | absolute growth rate |
| under continued observation | 1 | continue under active-surveillance |
| development or increase of peritumoral signal intensity (vasogenic oedema) | 1 | development or increase of oedema |
| gait disturbance | 1 | difficulty walking |
| discharged | 1 | discharged from active-surveillance |
| emotional state | 1 | emotional functioning |
| psychological burden | 1 | emotional functioning |
| exponential growth pattern | 1 | exponential growth pattern |
| exponential growth | 1 | exponential growth pattern |
| fatigue | 2 | fatigue |
| >15% | 1 | growth of tumor |
| >8.2% | 1 | growth of tumor |
| 50% increase in tumor volume | 1 | growth of tumor |
| any growth | 1 | growth of tumor |
| between 25% and 50% increase in tumor volume | 1 | growth of tumor |
| change in tumor volume | 1 | growth of tumor |
| clinically stable but had radiological progression | 1 | growth of tumor |
| growth | 7 | growth of tumor |
| more than 15% … increase in tumor volume | 1 | growth of tumor |
| more than 8.2% … increase in tumor volume | 1 | growth of tumor |
| overall tumor growth in cm3 | 1 | growth of tumor |
| progression | 2 | growth of tumor |
| radiographic progression | 1 | growth of tumor |
| significant growth | 1 | growth of tumor |
| significant tumor growth | 1 | growth of tumor |
| total growth | 1 | growth of tumor |
| tumor growth | 4 | growth of tumor |
| tumor progression | 1 | growth of tumor |
| volume changes | 1 | growth of tumor |
| volume growth | 1 | growth of tumor |
| meningioma volume | 1 | growth of tumor to greater than 10cm3 |
| radiological progression-free survival | 1 | growth-free survival |
| tumor progression-free survival | 1 | growth-free survival |
| hemiplegia | 1 | limb paralysis |
| motor deficit | 2 | limb weakness |
| linear growth pattern | 1 | linear growth pattern |
| linear growth | 2 | linear growth pattern |
| size (2D) | 1 | maximum 2D size of the tumor |
| diameter | 1 | maximum diameter of the tumor |
| final tumor diameter of the equivalent sphere in cm | 1 | maximum diameter of the tumor |
| maximum linear diameter | 1 | maximum diameter of the tumor |
| maximum tumour diameter | 1 | maximum diameter of the tumor |
| size (1D) | 1 | maximum diameter of the tumor |
| size of the tumor | 1 | maximum diameter of the tumor |
| tumor diameter | 2 | maximum diameter of the tumor |
| tumor size | 7 | maximum diameter of the tumor |
| meningioma-specific mortality | 1 | meningioma-specific mortality |
| intervention recommended | 1 | need for any treatment |
| intervention | 1 | need for any treatment |
| received intentional intervention for a preventative reason | 1 | need for any treatment |
| symptomatic progression resulting in treatment | 1 | need for any treatment |
| cerebrospinal fluid diversion | 1 | need for cerebrospinal fluid diversion |
| external beam fractionated radiotherapy | 2 | need for radiotherapy |
| fractionated radiotherapy | 1 | need for radiotherapy |
| radiation | 1 | need for radiotherapy |
| radiation therapy | 1 | need for radiotherapy |
| radiotherapy | 2 | need for radiotherapy |
| treated with radiotherapy | 1 | need for radiotherapy |
| gamma knife | 1 | need for stereotactic radiosurgery |
| gamma knife radiosurgery | 1 | need for stereotactic radiosurgery |
| GKS | 1 | need for stereotactic radiosurgery |
| radiosurgery | 2 | need for stereotactic radiosurgery |
| radiosurgical management | 1 | need for stereotactic radiosurgery |
| stereotactic radiosurgery | 3 | need for stereotactic radiosurgery |
| microsurgery | 1 | need for surgery |
| operated | 1 | need for surgery |
| resection | 1 | need for surgery |
| surgery | 11 | need for surgery |
| surgical excision | 1 | need for surgery |
| surgical management | 1 | need for surgery |
| surgical removal | 1 | need for surgery |
| surgical resection | 2 | need for surgery |
| surgically removed | 1 | need for surgery |
| treated surgically | 1 | need for surgery |
| surgery + radiotherapy | 1 | need for surgery and radiotherapy |
| surgery and radiation | 1 | need for surgery and radiotherapy |
| mental deterioration | 1 | neurocognitive functioning |
| neurocognitive functioning | 1 | neurocognitive functioning |
| neurocognitive variables | 1 | neurocognitive functioning |
| a cerebellar sign | 1 | neurological signs |
| ataxia | 1 | neurological signs |
| cerebellar deficit | 1 | neurological signs |
| development of new neurological deficit | 1 | neurological signs |
| neurologic progression | 1 | neurological signs |
| neurological functioning | 1 | neurological signs |
| ophthalmoplegia | 1 | neurological signs |
| tumor-attributed new neurological deficits | 1 | neurological signs |
| appearance of symptoms | 2 | new symptoms |
| brain tumor and treatment related symptoms | 1 | new symptoms |
| development of new neurological symptoms | 1 | new symptoms |
| new clinical symptoms | 1 | new symptoms |
| newly developed or worsening neurologic symptoms | 1 | new symptoms |
| number of patients becoming symptomatic | 1 | new symptoms |
| symptom development | 1 | new symptoms |
| symptomatic | 4 | new symptoms |
| symptomatic from an enlarging tumor | 1 | new symptoms |
| tumor-related symptoms | 1 | new symptoms |
| local tumor control | 2 | no tumor growth |
| stable | 1 | no tumor growth |
| stable clinically and radiologically | 1 | no tumor growth |
| tumor stability | 1 | no tumor growth |
| mortality unrelated to the meningioma | 1 | non-meningioma-specific mortality |
| non-meningioma-specific mortality | 1 | non-meningioma-specific mortality |
| were not able to undergo surgery | 1 | not able to undergo treatment |
| global HRQoL | 1 | Overall quality of life |
| HRQoL | 1 | Overall quality of life |
| patients' Quality of Life | 1 | Overall quality of life |
| died | 9 | overall survival |
| overall mortality | 1 | overall survival |
| overall survival | 1 | overall survival |
| parabolic | 1 | parabolic growth pattern |
| refused surgery | 1 | patient declined treatment |
| refused treatment | 1 | patient declined treatment |
| intervention as per patient request | 1 | patient request for treatment |
| performance status | 1 | physical functioning |
| physical functioning | 1 | physical functioning |
| (5-) and 10-year progression-free survival | 1 | progression-free survival |
| 10-year progression-free survival | 1 | progression-free survival |
| neurological progression-free survival | 1 | progression-free survival |
| progression-free interval | 1 | progression-free survival |
| progression-free survival | 7 | progression-free survival |
| progression-free survival rates at ... 5 years | 1 | progression-free survival |
| progression-free survival rates at 4 ... years | 1 | progression-free survival |
| quasi-exponential growth | 1 | quasi-exponential growth pattern |
| absolute growth | 1 | relative growth rate |
| fast growing | 1 | relative growth rate |
| medium growth | 1 | relative growth rate |
| no growth | 1 | relative growth rate |
| percentage of growth | 2 | relative growth rate |
| proportional annual growth rate (PGR) | 1 | relative growth rate |
| relative growth rate | 5 | relative growth rate |
| proportional growth rate per time | 1 | relative growth rate |
| relative growth rates … for maximum initial diameter (RGD) | 1 | relative growth rate |
| relative growth rates … for volume (RGV) | 1 | relative growth rate |
| slow growth | 2 | relative growth rate |
| focal seizures | 1 | seizure |
| seizure | 4 | seizure |
| sigmoid/self-limiting growth | 1 | sigmoid growth pattern |
| missing the window for stereotactic radiosurgery | 1 | stereotactic radiosurgery no longer a treatment option |
| remained asymptomatic | 2 | symptom-free |
| clinical progression but remained radiologically stable | 1 | time to clinical progression |
| time to the appearance of neurological symptoms | 1 | time to clinical progression |
| time to growth | 1 | time to growth |
| both clinical and radiological progression | 1 | time to progression |
| failure of continuous observation | 1 | time to progression |
| time to Ob-F | 1 | time to progression |
| time-to-progression | 1 | time to progression |
| 5-year tumor doubling time (5y-TdT) | 1 | tumor doubling time |
| tumor doubling time | 6 | tumor doubling time |
| volume doubling time (VDT) | 1 | tumor doubling time |
| tumor invasion into nearby sinuses | 1 | tumor invasion into venous sinus |
| venous sinus invasion | 1 | tumor invasion into venous sinus |
| continuous reduction | 1 | tumor regression |
| regressed | 1 | tumor regression |
| tumor regression | 1 | tumor regression |
| blind | 1 | visual impairment |
| visual deficit | 2 | visual impairment |
| vasogenic oedema | 1 | volume of edema |
| ellipsoid volume | 1 | volume of tumor |
| final tumor volume in cm3 | 1 | volume of tumor |
| segmentation volumetric analysis (SVA) | 1 | volume of tumor |
| tumor volume | 9 | volume of tumor |
| volume | 2 | volume of tumor |
| volumetric analysis | 2 | volume of tumor |
| volumetric analysis of tumor size | 1 | volume of tumor |
| volumetric measurements of tumor | 1 | volume of tumor |
| volumetric measurements of tumor and (edema) | 1 | volume of tumor and edema |
| aggravation of existing symptoms | 1 | worsening symptoms |
| progression of their symptoms | 1 | worsening symptoms |
| **178** | **267** | **53** |

**Supplementary Appendix 4 - Standardised Outcome Terms and Reporting Frequency**

| **COMET Core Area** | **COMET Outcome Domain & No.** | **Standardised outcome term** | **Reporting frequency** | **No. Defined** |
| --- | --- | --- | --- | --- |
| **Death** | Mortality/survival (1) | growth-free survival | 2 | 0 |
|  |  | meningioma-specific mortality | 1 | 0 |
|  |  | non-meningioma-specific mortality | 2 | 0 |
|  |  | overall survival | 11 | 5 |
|  |  | progression-free survival | 13 | 9 |
| **Physiological/clinical** | Eye outcomes (7) | visual impairment | 3 | 1 |
|  | General outcomes (9) | fatigue | 2 | 0 |
|  | Nervous system outcomes (17) | absolute growth rate | 18 | 9 |
|  |  | development or increase of oedema | 1 | 1 |
|  |  | difficulty walking | 1 | 0 |
|  |  | exponential growth pattern | 2 | 0 |
|  |  | growth of tumor | 30 | 17 |
|  |  | growth of tumor to greater than 10cm3 | 1 | 1 |
|  |  | limb paralysis | 1 | 0 |
|  |  | limb weakness | 2 | 0 |
|  |  | linear growth pattern | 3 | 0 |
|  |  | maximum 2D size of the tumor | 1 | 1 |
|  |  | maximum diameter of the tumor | 15 | 4 |
|  |  | need for any treatment | 4 | 0 |
|  |  | need for cerebrospinal fluid diversion | 1 | 0 |
|  |  | need for radiotherapy | 8 | 0 |
|  |  | need for stereotactic radiosurgery | 9 | 0 |
|  |  | need for surgery | 21 | 0 |
|  |  | need for surgery and radiotherapy | 2 | 0 |
|  |  | neurological signs | 8 | 2 |
|  |  | new symptoms | 14 | 2 |
|  |  | no tumor growth | 5 | 4 |
|  |  | parabolic growth pattern | 1 | 1 |
|  |  | quasi-exponential growth pattern | 1 | 1 |
|  |  | relative growth rate | 17 | 11 |
|  |  | seizure | 5 | 0 |
|  |  | sigmoid growth pattern | 1 | 0 |
|  |  | symptom-free | 2 | 0 |
|  |  | time to clinical progression | 2 | 1 |
|  |  | time to growth | 1 | 1 |
|  |  | time to progression | 4 | 2 |
|  |  | tumor doubling time | 8 | 1 |
|  |  | tumor invasion into venous sinus | 2 | 0 |
|  |  | tumor regression | 3 | 2 |
|  |  | volume of edema | 1 | 0 |
|  |  | volume of tumor | 18 | 0 |
|  |  | volume of tumor and edema | 1 | 0 |
|  |  | worsening symptoms | 2 | 0 |
| **Life impact** | Physical functioning (25) | physical functioning | 2 | 0 |
|  | Emotional functioning/wellbeing (28) | emotional functioning | 2 | 0 |
|  | Cognitive functioning (29) | neurocognitive functioning | 3 | 0 |
|  | Global Quality of Life (30) | overall quality of life | 3 | 0 |
|  | Delivery of care (32) | continue under active-surveillance | 1 | 0 |
|  |  | discharged from active-surveillance | 1 | 0 |
|  |  | not able to undergo treatment | 1 | 0 |
|  |  | patient declined treatment | 2 | 1 |
|  |  | patient request for treatment | 1 | 0 |
|  |  | stereotactic radiosurgery no longer a treatment option | 1 | 0 |
|  | **9** | **53** | **267** | **77** |

**Supplementary Appendix 5 - Types of Standardised Outcome Terms**

| **COMET Core Area** | **Binary event/time to event** | **Composite** | **Multiple category event**  **Clinician Reported** | **Multiple category event**  **Patient Reported** | **Multi-dimensional**  **health measures** |
| --- | --- | --- | --- | --- | --- |
| ***Death*** | meningioma-specific mortality | growth-free survival |  |  |  |
|  | non-meningioma-specific mortality | progression-free survival |  |  |  |
|  | overall survival |  |  |  |  |
| ***Physiological/clinical*** | symptom-free |  | maximum diameter of the tumor | visual impairment |  |
|  | no tumor growth |  | maximum 2D size of the tumor | difficulty walking |  |
|  | growth of tumor to greater than 10cm3 |  | volume of tumor | limb paralysis |  |
|  | tumor invasion into venous sinus |  | volume of edema | limb weakness |  |
|  | time to growth |  | volume of tumor and edema | fatigue |  |
|  | time to clinical progression |  | growth of tumor | seizure |  |
|  | time to progression |  | tumor regression | worsening symptoms |  |
|  | tumor doubling time |  | absolute growth rate | new symptoms |  |
|  | need for any treatment |  | relative growth rate |  |  |
|  | need for cerebrospinal fluid diversion |  | parabolic growth pattern |  |  |
|  | need for radiotherapy |  | exponential growth pattern |  |  |
|  | need for stereotactic radiosurgery |  | linear growth pattern |  |  |
|  | need for surgery |  | quasi-exponential growth pattern |  |  |
|  | need for surgery and radiotherapy |  | sigmoid growth pattern |  |  |
|  |  |  | development or increase of oedema |  |  |
|  |  |  | neurological signs |  |  |
| ***Life impact*** | continue under active-surveillance |  |  |  | physical functioning |
|  | discharged from active-surveillance |  |  |  | emotional functioning |
|  | patient request for treatment |  |  |  | neurocognitive functioning |
|  | patient declined treatment |  |  |  | overall quality of life |
|  | not able to undergo treatment |  |  |  |  |
|  | stereotactic radiosurgery no longer a treatment option |  |  |  |  |

**Supplementary Appendix 6 - Measurement of Standardised Outcome Terms**

| **Multiple category event**  **Clinician Reported** | **Indicators and/or tool(s) identified (deduplicated)** |
| --- | --- |
| maximum diameter of the tumor | Calculation (n=3), Radiological measurement (Modality not specified), Radiological measurement (MRI), Radiological measurement (CT or MRI) each with different methods |
| maximum 2D size of the tumor | Radiological measurement (CT or MRI) |
| volume of tumor | Radiological measurement (Modality not specified), Radiological measurement (CT or MRI), Radiological measurement (MRI) |
| volume of edema | Radiological measurement (CT or MRI) |
| volume of tumor and edema | Radiological measurement (CT or MRI) |
| growth of tumor | Calculation |
| tumor regression | Radiological assessment as per RANO criteria |
| absolute growth rate | Calculation |
| relative growth rate | Calculation |
| parabolic growth pattern | Calculation - Not specified |
| exponential growth pattern | Calculation |
| linear growth pattern | Calculation |
| quasi-exponential growth pattern | Calculation - Not specified |
| sigmoid growth pattern | Calculation - Not specified |
| development or increase of oedema | Radiological measurement (MRI) |
| neurological signs | The Neurologic Functional Status Scale (NFSS) |
|  |  |
| **Multiple category event**  **Patient Reported** |  |
| visual impairment | Not specified |
| difficulty walking | Not specified |
| limb paralysis | Not specified |
| limb weakness | Not specified |
| fatigue | POMS, BFI |
| seizure | Not specified |
| worsening symptoms | Not specified |
| new symptoms | Brain Cancer Module 20 (BCM20) |
|  |  |
| **Multi-dimensional**  **health measures** |  |
| physical functioning | Barthel Index (BI), Karnofsky Performance Scale (KPS) |
| emotional functioning | Hospital Anxiety and Depression Scale (HADS) |
| neurocognitive functioning | List of tests specified (n=2) |
| overall quality of life | 36-Item Short Form Survey (SF-36), European Organization for the Research and Treatment of Cancer Quality of Life Questionnaire (EORTC QLQ C-30) (Items 29 & 30 only) |
